# Life cycle, Ecological Characteristics, and Control of *Trachys yanoi* (Coleoptera: Buprestidae), an Important Pest of *Zelkova serrata*

**DOI:** 10.3390/insects8020035

**Published:** 2017-03-24

**Authors:** Masashi Ohsawa

**Affiliations:** Yamanashi Forest Research Institute, 2290-1 Saisho-ji, Fujikawa-cho, Minamikoma-gun, Yamanashi 400-0502, Japan; oosawa-ujk@pref.yamanashi.lg.jp; Tel.: +81-556-22-8001

**Keywords:** leaf miner, *Zelkova serrata*, early leaf abscission, jewel beetle, insect pest

## Abstract

This study was conducted to elucidate the life cycle and the ecological characteristics of *Trachys yanoi* Y. Kurosawa, an important pest of *Zelkova serrata* (Thunb.) Makino. Life cycle, mortality rates in developmental stages, annual population dynamics, and early leaf abscission were investigated. Adults emerged from under the bark of *Zelkova* trees in April and fed on *Zelkova* leaves. Females laid 49 eggs on average, mainly in May and early June. Eggs hatched after 17 days, and the larvae fed inside the leaves. They developed through three instars. In July, leaves with the final stage of larvae were abscised. Four days after abscission, the larvae pupated. New adults eclosed from pupae seven days after pupation, and the adults emerged from abscised leaves after an additional two days. In total, 1650 adults emerged per 1 m^2^ of forest floor, resulting in a major population increase. The newly emerged adults fed on the remaining *Zelkova* leaves, compounding the damage. In October, adults overwintered under the tree bark. Mortality rates in the egg, larval, and pupal stages were 41%, 58%, and 31%, respectively. The mortality rate among overwintering individuals was 43%. Because only *Zelkova* leaves that were abscised in July contained the larvae, and because only a small number of beetles emerged from non-abscised, mined leaves, the removal of abscised leaves at nine-day intervals over period of early leaf abscission is a simple and effective way to control the beetle.

## 1. Introduction

Leaf miners are a group of insects whose larvae burrow into plant leaves and feed on their tissues. They belong to four orders: Diptera, Lepidoptera, Coleoptera, and Hymenoptera. Diptera and Lepidoptera have a large number of leaf miner species, in contrast with Coleoptera. Coleopteran leaf miners are concentrated in the three families: Bupresitidae, Chrysomelidae, and Curculionidae [1]. In the Bupresitidae, miners are placed in the subfamily Trachyinae and are primarily tropical in distribution and poorly investigated [1]. In fact, the majority of ecological research on leaf miners has addressed the order Lepidoptera [1,2,3]. Moreover, leaf miner research has been conducted mainly in North America [4,5,6] and Europe [7,8,9].

In the Trachyinae, *Brachys tessellantus* (Fabr.) and *B. ovatus* Web., buprestid leaf miners of oak trees in North America, have been studied on their life cycle, survivorship, and early leaf abscission [6,10,11,12,13]. Except for these species, studies addressing this subfamily, including Asian species, are quite limited because most Trachyinae species do not significantly damage trees.

An outbreak of the leaf miner *Trachys yanoi* Y. Kurosawa (Trachyinae, Buprestidae) is occurring throughout much of central Japan. In summer, the leaves of the host tree *Zelkova serrata* (Thunb.) Makino turn brown, and seriously damaged trees are completely defoliated. *T. yanoi* mines *Z. serrata* leaves in its larval stage, while adults feed on leaves directly. This beetle is the most serious forest pest among *Trachys* species in Japan, and its outbreaks have previously been recorded in two locations in Japan: in Kyoto [14,15,16] and Tokyo [17].

The genus *Trachys* is distributed widely, especially in tropical regions from Africa to Asia, and 20 species have been recorded in Japan [18]. All *Trachys* species found in Japan are leaf miners and ecologically different from *Brachys* species examined in North America in the number of instars, overwintering in adult stage, and the period staying in early abscission leaves.

*T. yanoi* is small (2.7–4.5 mm in length and 1.3–2.3 in width) with an ellipsoid body. Its dorsal side is reddish to dark brown, with whitish wavy zones on the elytra. This species is distributed throughout temperate East Asia (Japan, Korea, and China), and an outline of its life cycle has been described as follows [19]. After overwintering, adult beetles become active in April, feed on the leaves of *Zelkova* trees, and mate, after which females lay eggs on the surface of the leaves. The eggs hatch and larvae mine leaves and then feed on their tissues. Judging from larval size, larvae seem to develop through three instars [15]. After early leaf abscission (early leaf fall), the larvae pupate within the leaves, and new adults emerge from the now-dead leaves. These adults feed on leaves in the crown of *Zelkova* trees and then move to the underside of *Zelkova* bark in preparation for overwintering. This species has a 1-year life cycle and new adults emerge in summer.

*Zelkova serrata*, a host of *T. yanoi*, is a large deciduous tree attaining 40 m in height and 2 m in stem diameter [20]. It is distributed throughout the temperate zone in Japan, China, and Korea. In Japan, it is often planted on shrine grounds, in parks, in gardens as windbreaks, on mountains for timber production, and along roadsides. The valuable wood is used as a building material and for furniture.

Spraying insecticides is often impractical because spraying in high-elevation locations (tree crowns) is difficult and can result in the forest biodiversity being degraded. To control the beetle using appropriate environmentally sustainable methods, we must first understand its ecological characteristics in detail. The aims of this study were to elucidate the detailed life cycle and related ecology of *T. yanoi*, to determine methods for reducing the damage caused by the beetle.

## 2. Materials and Methods

### 2.1. Study Site

**Figure 1:**
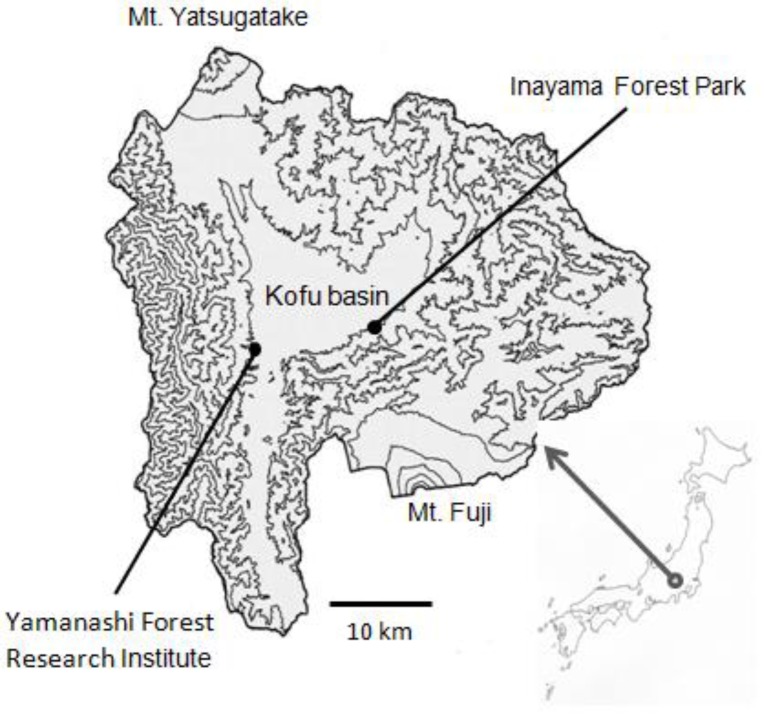
Study sites in Yamanashi Prefecture, Japan.

This study was conducted mainly in a forest stand in Inayama Forest Park, in Fuefuki City, Yamanashi Prefecture, Japan (35°35′14″ N, 138°39′5″ E; 630 m a.s.l.; Figure 1), where *T. yanoi* has caused severe damage; *Zelkova* trees have lost the majority of their leaves during late summer in most years. This stand is a seminatural forest dominated by *Z. serrata*, with an area of 2.4 ha. The *Zelkova* trees in this stand are approximately 150 years old, and on average, they are 90 cm in diameter at breast height (DBH) and 24 m in height. For the purposes of this study, this stand will be referred to as the Inayama forest.

*Zelkova* trees planted in an experimental forest of the Yamanashi Forest Research Institute in Fujikawa-cho, Yamanashi (35°33′38″ N, 138°26′44″ E, 320 m a.s.l.; Figure 1) were used for investigating survivorship and duration in oviposition, egg hatching, and larval growth. These trees sustained moderate damage from the beetle, losing about half of their leaves in late summer. They were 15 years old, and, on average, 3.7 cm in DBH and 4.1 m in height.

### 2.2. Annual Dynamics of T. yanoi Population

An aerial Malaise trap (flight-interception design; 196 cm long, 70 cm wide) was used to catch *T. yanoi* adults. The main body of the trap consisted of a rectangular cross-panel of netting with a triangular roof and bottom made of netting. Both top and bottom were attached to collecting bottles. Only the upper bottle containing 200 mL of propylene glycol as a preservative was used to catch beetles.

Three aerial Malaise traps, >50 m apart, were deployed in the Inayama forest in 2006, 2007, and 2008 to catch *T. yanoi* adults and observe population changes between seasons. Each trap was hung from a *Zelkova* tree branch at a height of 8.7 m above the ground, on average. Bottles were replaced once a month in 2006 and twice a month in 2007 and 2008. All beetles were removed from each bottle, and *T. yanoi* were sorted and counted.

The population of the overwintered adults was compared with that of the new adults which emerged in summer. The daily average number of individuals in May was used as the overwintered population, and the number in the period from mid-August to mid-September was used as the population of the new adults.

### 2.3. Developmental Stages of T. yanoi on/in Leaves on Zelkova Branches

A small branch (about 50 cm long) was chosen from each of three *Zelkova* trees, >35 m apart, in the Inayama forest and taken to the laboratory approximately every 15 days from April to November in 2009, 2010, and 2011. The numbers of leaves, mined leaves, and leaf scars of abscised leaves were enumerated on the three small branches. Subsequently, 20 leaves were randomly selected from each branch and examined to determine the number and conditions of eggs, larvae, and pupae on/in the leaves. When larvae were found, the width of the head and length of the body were measured.

These leaves were also examined for the approximate percentages of leaf areas mined by larvae and eaten by adults. The percentages were calculated based on the area data measured with a dot grid area estimation, or they were directly estimated by visual observation. The data for the percentages of leaf areas eaten by overwintered adult beetles and mined by larvae were taken just before early leaf abscission occurred (late June to early July). The data for the percentage of leaf areas eaten by new adults were taken just before overwintering started (mid-September). The numbers of early abscised leaves in the three branches were determined from abscised leaf scars on the branches just after early leaf abscission ended (mid-August to early September) and their percentages were calculated.

### 2.4. Developmental Stages of T. yanoi in Early Abscised Leaves on the Ground

Traps were set to collect abscised leaves (mesh boxes with an open upper part, 33 cm× 49 cm × 30 cm) on the ground near each of the aerial Malaise traps in the Inayama forest from April to November of 2009, 2010, and 2011. Abscised leaves were collected approximately every 15 days. Then, 20 abscised leaves were randomly selected from the traps, and the numbers of living and dead eggs, larvae, pupae, and adults of *T. yanoi* on/in these leaves were enumerated. 

The remaining abscised leaves were kept in paper bags at room temperature, and the adults that emerged from these leaves were counted. These leaves then were dried at room temperature, and subsequently in an oven at 80° for 48 h, and weighed.

### 2.5. Leaf Abscission and T. yanoi Emergence

Emergence traps were used to catch adult beetles that emerged from abscised leaves in the Inayama forest. The main body of the trap was a pyramid of netting with a 71 cm × 71 cm square base. The top of the 70 cm high pyramid was connected to a collecting bottle containing 200 mL propylene glycol as a preservative. At that time (25 July 2012) when most of mined leaves had abscised and the majority of the *T. yanoi* were still within the abscised leaves, four traps, 14.2 m apart on average, were set on the ground in the Inayama forest. Bottles were collected on 25 August of the same year, after all beetles had emerged; adult beetles caught in the bottles were counted. Then, the numbers of early abscised leaves under emergence traps were counted. The adult emergence per abscised leaf was calculated.

In total, 110 abscised leaves (30 from each site; 10 torn leaves were subsequently removed) were collected near the four trap sites on 1 August 2012, and examined for numbers and conditions of eggs, larvae, pupae, adults, exuviae, emergence holes, and mined parts on/in the leaves. Mortality rates of *T. yanoi* in the larval, pupal, and adult (before emergence from the leaves) stages were calculated as follows: [the number of the dead beetles in the developmental stage/(the number of the dead beetles in the stage + the number of the beetles which grew up to be its next stage)] × 100.

Some mined leaves remained on branches even after the early abscission periods. As these non-abscised, mined leaves would also contribute to the number of emerging adults, 120 non-abscised leaves were collected after the leaf abscission period was over (60 leaves on 28 July 2010 and 60 on 21 August 2013). The leaves were dissected and examined for numbers and conditions of eggs, larvae, pupae, adults, exuviae, and emergence holes on/in the leaves. Fifty non-abscised leaves were also collected with an emergence hole per each and measured the area of leaves and mined parts in them and calculated percentages of mined part.

### 2.6. Oviposition

On 5 April 2014, before *T. yanoi* began laying eggs, 13 beetle pairs mating on *Zelkova* leaves (consisting of one beetle mounted on the other) were collected in the Inayama forest, and each pair was kept in a glass cylinder (10 cm in diameter and 20 cm in height) with a mesh top and wet sand in the bottom. A *Zelkova* branch (about 20 cm long with approximately 20 leaves) was placed in a small bottle with water and kept in the cylinder as food for the beetles. Branches were replaced to avoid leaf wilt. Before each branch was replaced, the number of eggs laid on its leaves was counted. The number of eggs from pairs, in which both individuals survived until the end of June, was used to represent the number of eggs laid. The longevity of each beetle was also recorded.

### 2.7. Period Required for Eggs to Hatch, Larval Growth, and Adult Eclosion

In 2014, six small branches from *Zelkova* trees were selected in the experimental forest of the Yamanashi Forest Research Institute. A black ink mark was placed around any *T. yanoi* eggs that were found on the leaves of these branches. These eggs were laid on the leaves from 2 May to 16 June 2014. These marked eggs were observed, checking for hatching and larval growth. The six selected branches including the marked leaves were covered with mesh bags before early leaf abscission, and collected the abscised leaves. Each of these abscised leaves was placed in a Petri dish and adult emergence was observed. After all adults emerged, the leaves investigated were dissected and examined for numbers and conditions of larvae, pupae, adults, exuviae, and emergence holes on/in the leaves. Finally, the durations of the egg, larval, pupal, and adult stages were tallied, and the mortality rates of the egg and larval stages were calculated. The period of early leaf abscission was also observed.

Forty-seven early abscised leaves were collected immediately after they were abscised on 24 June 2014. Twenty-five of them were carefully dissected and the *T. yanoi* larvae were removed from inside the abscised leaves. Each larva was then placed into a Petri dish and its further growth was observed. The remaining 22 abscised leaves were placed into Petri dishes and adult emergence was obseved. The durations of larval, pupal, and adult stages after early leaf abscission were tallied.

### 2.8. Overwintering

*T. yanoi*’s overwintering location preferences (height and cardinal direction) on trunks of *Z. serrata* in the Inayama forest was explored. For height, all partially peeled barks on a 50 cm × 100 cm area of the trunk surface of a *Zelkova* tree were peeled and the number of overwintering *T. yanoi* under each area of bark was determined. This was done at five heights (2, 5, 7, 12, and 15.5 m) on the trunks of six *Zelkova* trees from the end of October 2007 through mid-March 2008. For cardinal direction, a strip of cloth (5 cm wide) was wrapped around the trunk of each of three *Zelkova* trees at 130 cm above the ground in early October 2006, and beetles overwintering under the cloth were counted in mid-March 2008 in each direction: north, northwest, west, southwest, south, southeast, east, and northeast. The numbers of *T. yanoi* overwintering were compared among the four heights and eight cardinal directions by a Kruskal-Wallis H test.

To determine the mortality rate in overwintering individuals, 30 pieces of half-peeled *Zelkova* bark were peeled back and all *T. yanoi*, dead or alive, that were overwintering under the bark on 10 April were removed just before the end of the overwintering period. These beetles were brought back to the laboratory and kept at room temperature. After the live beetles started moving, live and dead individuals were separated and the mortality rate was calculated. The causes of death, such as spider silks, wounds, a mass of fungal spores or mycelia, etc. were examined under a stereo microscope. When entomopathogenic fungi were observed on the surface of the dead beetle, further observation was carried out with a microscope.

## 3. Results

### 3.1. Annual Dynamics of T. yanoi Population

**Figure 2:**
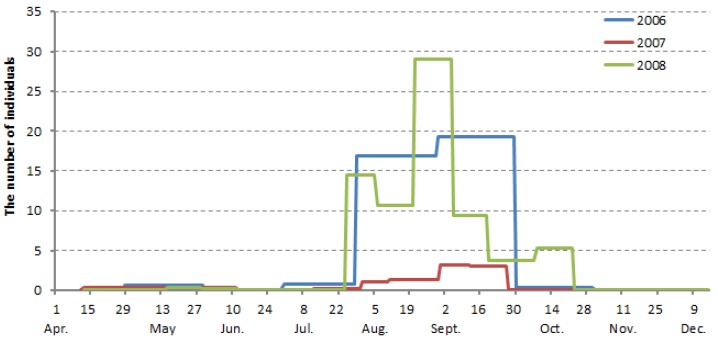
Seasonal abundance of *Trachys yanoi* adults caught by aerial Malaise traps in 2006, 2007, and 2008.

The numbers of beetles caught by the aerial Malaise traps in 2006, 2007, and 2008 are shown in Figure 2. Overwintered adults began to be caught in mid-April and disappeared after mid-June, indicating that the majority of them had died by that time. New adults began to emerge in late July and stayed active until late September to mid-October.

The number of the overwintered adults caught in traps was much smaller than that of the new adults caught later in the same year (Figure 2). On average, over three years, the number of new adults (the number caught from mid-August to mid-September) was approximately 33 times larger than that of the overwintered adults (the number caught in May).

### 3.2. Developmental Stages of T. yanoi on/in Leaves on Zelkova Branches

**Figure 3:**
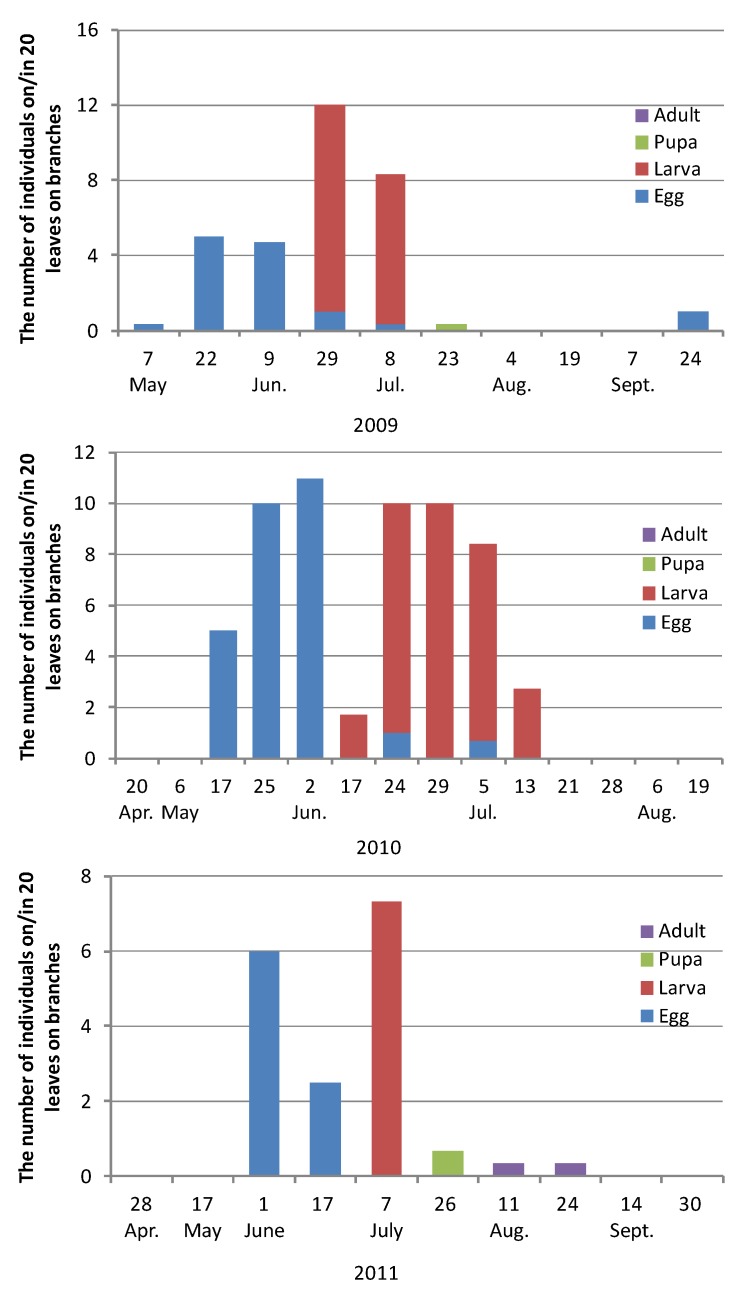
The numbers of *Trachys yanoi* in each developmental stage on/in leaves on branches in 2009, 2010, and 2011.

The numbers of *T. yanoi* eggs, larvae, pupae, and adults on/in leaves on *Zelkova* branches in 2009, 2010, and 2011 are shown in Figure 3. Overwintered adults emerged from under the partially peeled bark and began feeding on *Zelkova* leaves in late April, soon after *Zelkova* budburst. After feeding and mating, they laid eggs singly on the upper surface of *Zelkova* leaves, mainly in mid-May to mid-June. The eggs hatched in mid- to late June, and larvae developed rapidly through the three larval instars. The average larval head widths and body lengths were, respectively, 0.21 ± 0.07 mm and 1.9 ± 0.50 mm (*n* = 12), for first instar larvae, 0.32 ± 0.05 mm and 2.9 ± 0.73 mm (*n* = 43) for second instar larvae, and 0.54 ± 0.05 mm and 5.7 ± 1.17 mm (*n* = 54) for third instar larvae. The number of mined leaves on branches rapidly decreased because of early leaf abscission in July to early August. Most leaves with living miners were abscised and pupation occurred on non-abscised leaves only infrequently.

### 3.3. Developmental Stages in Early Abscised Leaves on the Ground

**Figure 4:**
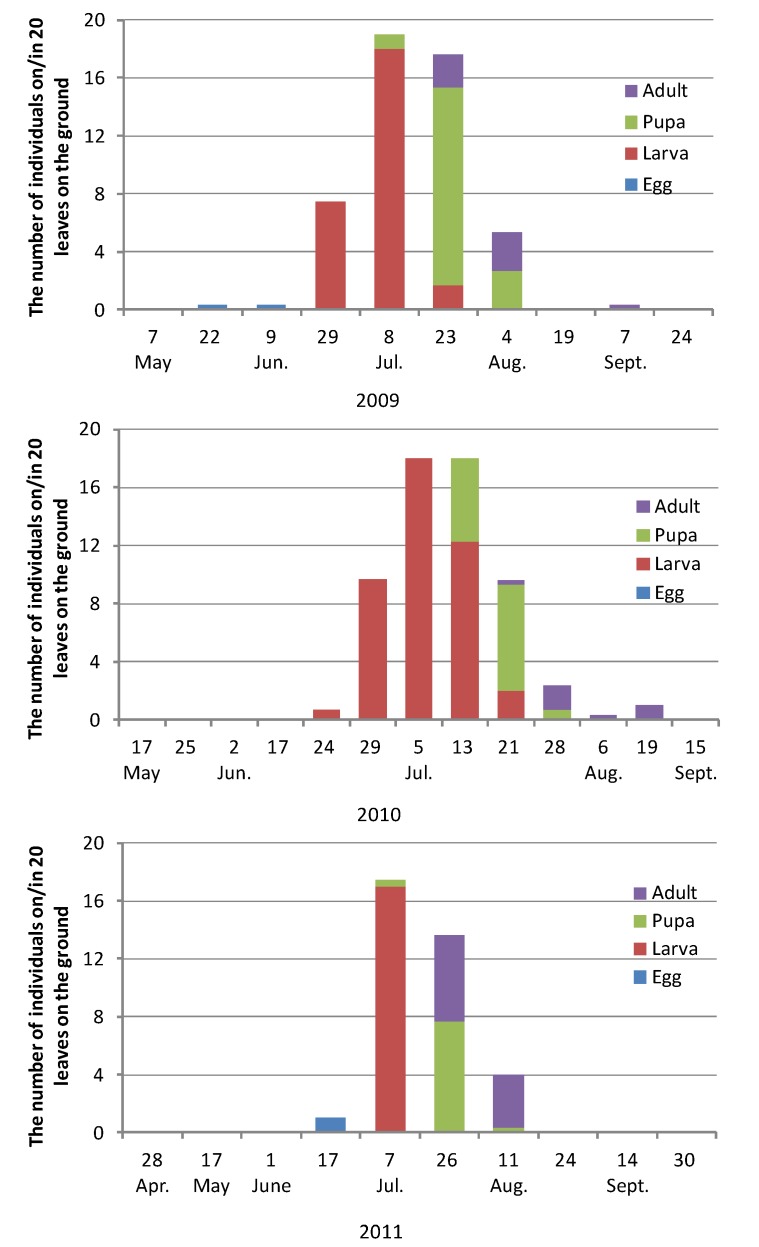
The numbers of *Trachys yanoi* in each developmental stage on/in abscised leaves in 2009, 2010, and 2011.

Figure 4 shows the results of abscised leaves in 2009, 2010, and 2011. Larvae in abscised leaves were found mainly from the end of June to late July and almost all were third instar larvae. Pupae were found mainly from mid-July to early August, and new adults emerged mainly from late July to early August. All of the eggs on abscised leaves had already hatched or died.

**Figure 5:**
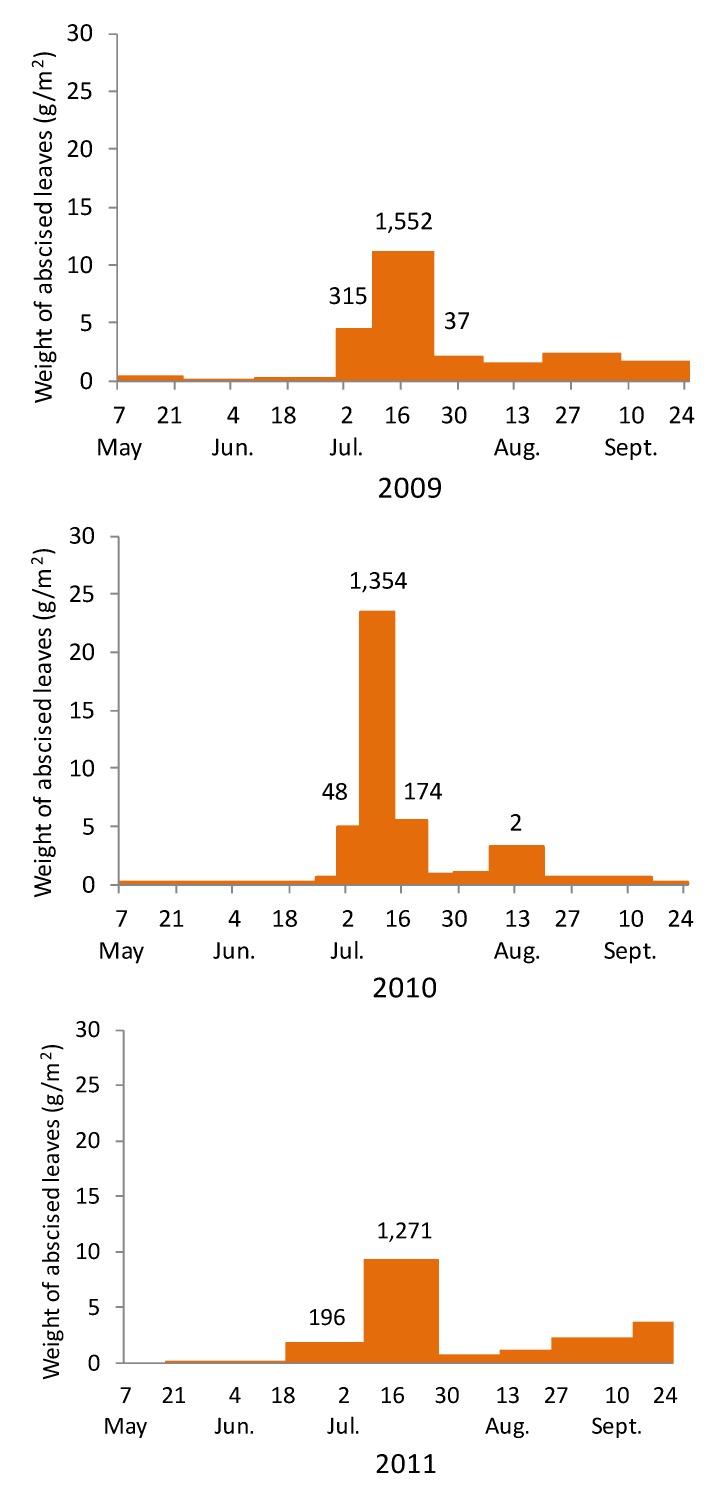
Weight of abscised *Zelkova serrata* leaves in 2009, 2010, and 2011, and the numbers of *Trachys yanoi* (above bars) that emerged from these leaves.

The weights of abscised leaves caught in traps during each period in 2009, 2010, and 2011 are shown in Figure 5. Many leaves fell in July, especially in the period from 1 July to 25 July, which resulted from the early leaf abscission caused by mining. Many adult beetles emerged from leaves abscised in this period after being kept in laboratory. The numbers of emerged adults per 1 m^2^ of forest floor are shown above bars in Figure 5. The number was 1650 per 1 m^2^ on three-year average. No adult beetles emerged from the minority of leaves that were abscised in the other periods from spring to autumn (Figure 5).

### 3.4. Leaf Abscission and T. yanoi Emergence

Using emergence traps on the ground of the Inayama forest, on average, the number of emerged adults was approximately 36.5% of the number of early abscised leaves. This shows that a relatively high ratio of the beetles survived on the ground and successfully emerged from the abscised leaves in a natural condition in 2012, considering that the emergence traps do not catch all adults emerged.

In 110 early abscised leaves collected near the four emergence trap sites, most adults had already emerged, and dead larvae, pupae, adults, and exuviae remained in the mines of these abscised leaves. The percentages of leaves with one, two, or three mined parts were 46.4%, 42.7%, and 10.9% of all abscised leaves, respectively. Only one adult emerged from each mined leaf at best, and mortality rates were 58.6% for the larval stage, 30.7% for the pupal stage, and 2.6% for the adult stage (before emergence from the mined leaves; Table 1). Living pupae in abscised leaves accounted for 17% of the total pupae. Because of their blackish body color, they were considered to be present just before the eclosion of adults. Because the calculation of mortality assumed that all living pupae could develop into adults, the mortality rates in the pupal (30.7%) and adult (2.6%) stages in Table 1 may change slightly if some of them cannot emerge.

Non-abscised, mined leaves made up 23.8% of all mined leaves on average in the Inayama forest. However, most *T. yanoi* died before the pupal stage in non-abscised, mined leaves. Only 5.8% of non-abscised, mined leaves showed signs of adult emergence, such as emergence holes or pupal exuviae. On the other hand, adults emerged from 36.5% of abscised, mined leaves. Thus, although some adults can emerge from mined but non-abscised leaves remaining on branches, the infrequency of such emergence events makes them unimportant.

All of the larvae in 25 early abscised leaves that were collected just after abscission were in the third instar. After being removed from the leaves, they remained stationary in Petri dishes. These larvae pupated after 4.1 ± 0.8 days, and adult eclosion occurred 7.1 ± 0.4 days after pupation. The new adults became active 2.0 ± 0.5 days after the eclosion. A total of 13.2 days were needed from early abscission to adult emergence (active adult).

Adults emerged 12.8 days after abscission from 22 early abscised leaves that were placed inside Petri dishes.

### 3.5. Oviposition, Egg Hatch, and Larval Growth of T. yanoi

**Figure 6:**
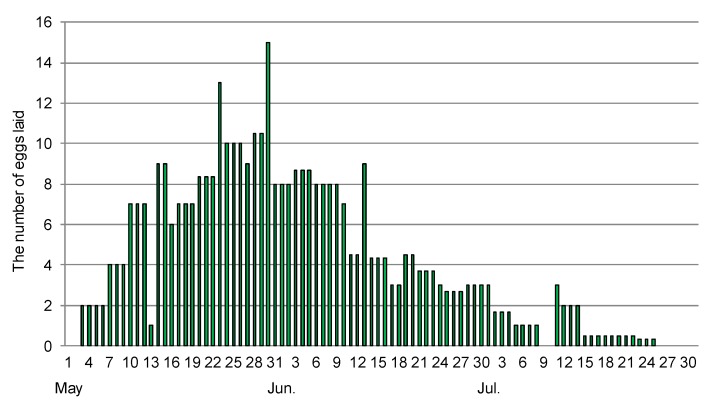
The number of eggs laid by eight pairs of *Trachys yanoi*.

Eight pairs of beetles survived in breeding condition and laid eggs on *Zelkova* leaves at room temperature between 9 May and 25 August (Figure 6). The number of eggs laid in this breeding period was, on average, 49.2 (25–74 eggs) per pair. In this study, overwintered adults under artificial conditions decreased in number from May to November. Half of them survived until the end of August and one adult survived until 2 December.

**Figure 7:**
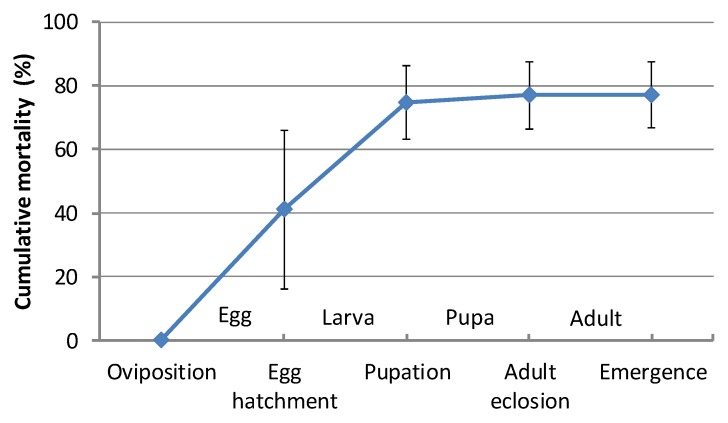
Cumulative mortality (%, average + SD) of *Trachys yanoi* in each stage of life cycle.

Six hundred and sixty-one eggs were found in the experimental forest and their development was observed. Eggs hatched between 19 May and 16 June, taking an average of 16.5 ± 4.8 days (*n* = 345) to hatch. Additionally, an average of 24.5 ± 5.5 days (*n* = 246) were required between egg hatching and early leaf abscission. Finally, the average length of the period from early leaf abscission to adult emergence was 11.7 ± 2.7 days (*n* = 145). The mortality rates of eggs, larvae, pupae, and adults before emergence were 41.1%, 56.7%, 8.8%, and 3.4%, respectively (Figure 7). The period of early leaf abscission was 31 days from 18 June to 18 July.

### 3.6. Overwintering

The beetles overwintered at all heights investigated on *Zelkova* trunks (Table 2). More beetles seemed to overwinter in lower parts (≤7 m) than in higher parts. However, the differences among heights were not significant (*p* > 0.05; Kruskal-Wallis H test). No orientation preference was noted for overwintering (*p* > 0.05; Kruskal-Wallis H test, Table 3).

A small Japanese cypress [*Chamaecyparis obtusa* (Sieb. et Zucc.) Endl.] forest exists next to the Inayama *Zelkova* forest. Many *T. yanoi* individuals were found to have overwintered under the partially peeled bark of Japanese cypress trees, indicating that the beetles can overwinter under the bark of other tree species.

From under 30 partially peeled areas of bark on *Zelkova* trees at the end of the overwintering period, 1634 overwintering individuals, including dead ones, were collected. During overwintering under the bark, 37.3% ± 21.4% of all beetles collected were dead. The causes of these deaths were attributed to *Beauveria bassiana* (Bals.-Criv.) Vuill. (an entomopathogenic fungus, 11.6%), other fungi (2.6%), spiders (0.3%), and unknown (28.0%).

### 3.7. Damage to Zelkova Trees

*T. yanoi* causes damage to *Zelkova* trees at three stages of its life cycle. First, in spring, the adult beetles feed on *Zelkova* leaves after overwintering. Fifteen percent of the leaf area (average of three years) was eaten. The damage caused during this period was relatively small. Second, *T. yanoi* larvae extensively mine within *Zelkova* leaves. The mined parts of the leaves accounted for 10% of the whole leaf on average. When *Zelkova* trees abscised the larva-afflicted leaves in early July, the extent of the damage was readily visible. The number of early abscised leaves accounted for an average of 65% of all leaves. Finally, newly emerged *T. yanoi* adults fed on the remaining *Zelkova* leaves, compounding the damage. The eaten parts accounted for 70% (including 15% by overwintered adults) of the remaining leaf area on average and severely eaten leaves were abscised. Thus, *Zelkova* trees in the Inayama forest frequently lost almost all leaves by the end of summer.

## 4. Discussion

### 4.1. Estimation of the Life Cycle from the Results of This Study

Based on the results in this study, the life cycle of *T. yanoi* can be described as follows. Adult *T. yanoi* overwinters under the partially peeled bark of *Zelkova* trees or other nearby trees, emerging just after bud burst in late April. Adults feed on *Zelkova* leaves after emergence. Eventually they mate and lay eggs singly on the upper surfaces of leaves from April to June, with peak oviposition occurring in May.

Female *T. yanoi* laid 25–73 eggs (49 on average) under artificial breeding conditions. The mortality rate in the egg stage was 41.1%. As *T. yanoi* eggs lack a hard shell and seem poorly protected, they may be vulnerable to both physical damage and predators. Eggs hatched after 17.0 days on average. Eggs laid late in the oviposition period hatched after 6–10 days, but those laid earlier required more time, with some taking more than 30 days to hatch.

*T. yanoi* larvae commence mining the mesophyll tissue of *Zelkova* leaves in the area under the eggs. Judging from the numbers of exuviae remaining within individual mines and changes in larval size, larvae were estimated to develop through three instars. Early leaf abscission occurs in July, when the larvae are mainly in the final instar. Mortality rates in the larval stage differed by location: 56.7% in the experimental forest of the Yamanashi Forest Research Institute in 2013 and 58.6% in the Inayama forest in 2012. The rate was 27.5% in the experimental forest of Yamanashi Forest Research Institute in 2012 [21]. Because, generally, only one larva can survive in a given leaf, the number of eggs on one leaf strongly affects the larval mortality rate. In Inayama forest, there were a number of leaves mined by two or three larvae. Moreover, I found some leaves that had six mining larvae. Thus, resource competition in the larval stage can be an important factor for the mortality of this beetle. Moreover, adults of the beetle also eat leaves of *Zelkova* trees. They feed on leaves on which their eggs were laid, although they do not feed on the leaf parts around their eggs. Resource competition between the larval and adult stages can occur if the population increases. Mortality rates were 41.1% for eggs, 57.7% (average of 56.7% and 58.6%; see above) for larvae, 30.7% for pupae, and 2.6% for adults before emergence. However, investigations in the pupal and adult stages in Petri dishes produced very different results, especially in the pupal stage: mortality rates were 8.8% for pupae and 3.4% for adults before emergence. Investigations in the laboratory may underestimate the mortality because they are placed in artificial conditions with far fewer natural enemies and less environmental disturbance.

Overwintered adults were neither caught by traps nor observed in the field after mid-June; i.e., most of them died. However, an investigation of oviposition showed that they could survive much longer under artificial conditions. Half could survive until the end of August. Even under natural conditions, it is possible that a small number of overwintered beetles may survive and live together with the new adults in summer. Two eggs were found in September in 2009 (Figure 3); this is very rare and it could not be clarified which adults (overwintered or new ones) had laid them.

Soon after early leaf abscission, larvae pupate within the mines, and new adults emerge from the dead leaves. It took 13.2 days from early leaf abscission to adult emergence in the Inayama forest and 11.7 days in field observations in the experimental forest. Because the branches on which the leaves were investigated were covered with mesh bags before early leaf abscission in the field observations, mined leaves may have been protected from physical disturbances such as wind or raindrops, causing leaf abscission to be delayed, in which case the duration between early leaf abscission and adult emergence may have become shorter. On average, 54.2 days were required for *T. yanoi* to develop from oviposition to adult emergence.

Non-abscised leaves comprised 23.8% of all mined leaves in the Inayama forest. These leaves probably did not abscise because the *T. yanoi* within them died in the larval stage, and there were therefore insufficient stimuli for early leaf abscission. Only 5.8% of non-abscised, mined leaves showed signs of adult emergence, such as emergence holes or pupal exuviae. In contrast, adults emerged from 36.5% of abscised, mined leaves.

New adults remain briefly on the leaves of understory vegetation, but soon fly up to the crowns of *Zelkova* trees and feed on the leaves. In the Inayama forest, an average of 1650 adults emerged per 1 m^2^ of forest floor (Figure 5). In October, adult *T. yanoi* move under the partially peeled barks of *Zelkova* or other trees and overwinter there.

In the three years of observation, the population increased by more than 33-fold its previous size (overwintered population). Aerial Malaise traps catch more beetles when the beetles are highly active. Overwintered adults may be less active in spring than the new adults are in summer, which may have affected the number of beetles caught in the aerial Malaise traps. However, field observations also indicated that *T. yanoi* increased in number as they moved from the overwintered adults to the new adults, although the difference in the population size may be less than 33-fold. In summer to autumn, some beetles may die because of a lack of *Zelkova* leaves, while some leave the area to find *Zelkova* leaves. The mortality rate of *T. yanoi* overwintering under the *Zelkova* barks was 37.3%, with *Beauveria bassiana* identified as the main cause. The population may also decrease during overwintering because some adults may not be able to obtain safe locations, such as under the bark. Thus, *T. yanoi* populations display a cyclical pattern of summer population increases, as the new adults emerge and temporarily thrive, and decrease in number from autumn to the following spring.

### 4.2. Control of the Beetle

*T. yanoi* lives in the relatively inaccessible crowns of large *Zelkova* trees for much of its life cycle, confounding normal control methods. However, *T. yanoi* descends from the *Zelkova* forest canopy at two times during its life cycle: when late-instar larvae fall to the ground with abscised *Zelkova* leaves, and when adults overwinter under partially peeled *Zelkova* bark on the main trunk. Removing early abscised *Zelkova* leaves can be an effective means of controlling *T. yanoi* because most infested leaves fell to the ground over a period of approximately one month (1 July to 25 July in the Inayama forest, 18 June to 18 July in the experimental forest). The majority of beetles emerged from abscised leaves 9.0 (11.7 − 2.7) to 14.1 (13.2 + 0.9) days after leaf abscission. The timing of early leaf abscission fluctuates to some extent because it may be affected by physical disturbances such as wind or rain. Therefore, *Zelkova* leaves abscised should be removed at least three times at approximately nine-day intervals over the period of early leaf abscission (one month). Although some beetles emerge from non-abscised leaves that remain on the tree, these are a decided minority.

This approach is more effective for isolated *Zelkova* trees growing in gardens, shrines, yards, etc., because the beetles can move in from nearby *Zelkova* trees. Because the timing of leaf abscission varies with climate, to apply this method it is important to know the exact period of leaf abscission in a target location and its interannual fluctuation.

Tsuchiya sprayed early abscised leaves with insecticide under artificial experimental conditions and showed that the insecticide reduced emergence of *T. yanoi* [17]. Spraying early abscised leaves on the forest floor with insecticide may be seen as an option if the timing and frequency are appropriate.

The *T. yanoi* population size changed year by year. For example, the number of *T. yanoi* was small in 2007 compared with numbers in 2006 and 2008 (Figure 2). To find a more effective way to control this insect pest, more work, such as long-term monitoring of the beetle population, is needed to clarify the patterns and causes of interannual fluctuations in population size.

Some death in larvae in mines and adults overwintering was attributable to parasitoid wasps and *B. bassiana*, respectively. Intensive research on the biological control using these natural enemies is needed.

The genus *Trachys* is distributed widely from Africa to Asia. Considering that *Trachys* spp. are leaf feeders in both their larval and adult stages, and that they are able to increase their population rapidly, they have the potential to be significant insect pests, especially in tropical regions where many *Trachys* species are found [1]. *Trachys minutus* (L.), which is endemic to Europe and Asia, was recently found in Massachusetts, US, as an introduced species [22]. Such alien invasive species may cause serious damage to native or introduced vegetation. A leaf miner, *Cameraria ohridella* Deschka & Dimič, has invaded locations in Europe and caused damage to horse chestnut trees, *Aesculus hippocastanum* L. [23,24,25].

This study showed that early leaf abscission provides an opportunity to reduce the population of *T. yanoi* by removing early abscised leaves. However, it also showed that the timing and frequency of this application is important because leaf abscission occurs during a rather short period (one month) and the beetles become adults and emerge from abscised leaves in nine to 14 days. This method can be used to develop further control methods for other leaf miners, especially *Trachys* spp.

## 5. Conclusions

This study elucidated the life cycle, mortality rates, annual population dynamics of *T. yanoi*, and early leaf abscission of *Z. serrata* in detail, to control this beetle miner using appropriate environmentally sustainable methods. Because only *Zelkova* leaves that are abscised in mid-summer contained the larvae, and because the adults emerge from abscised leaves in, at least, nine days, the removal of abscised leaves at nine-day intervals over period of early leaf abscission in mid-summer (during ca. one month) is a simple and effective way to control this beetle.

## Figures and Tables

**Table 1:** The numbers of dead larvae, dead pupae, and dead adults of *Trachys yanoi* in early abscised leaves, and their respective mortality rates (%).

Sites	Examined Leaves	Mines	Dead Larvae	Dead Pupae	Pupae	Dead Adults	Adult Emergence *
No.1	29	53	32	6	3	1	11
No.2	28	46	26	9	4	0	7
No.3	29	45	23	4	2	0	16
No.4	24	37	25	4	4	0	4
Total	110	181	106	23	13	1	38
Mortality rate	-	-	58.6	30.7	-	2.6	

* The presence of emergence hole.

**Table 2:** The numbers per 100 cm^2^ (median and quartile deviation) of *Trachys yanoi* overwintering at each height of *Zelkova* tree trunks.

Height (m)	No. of Beetles/100 cm^2^	
	Median	Quartile Deviation
2	2.68	2.17
7	2.03	0.72
12	1.08	0.41
15–16	1.68	0.80

**Table 3:** The proportions (%, median and quartile deviation) of *Trachys yanoi* individuals overwintering in each cardinal direction on *Zelkova* tree trunks.

Cardinal Direction	Proportion (%)	
	Median	Quartile Deviation
Southeast	12.79	2.50
South	4.31	9.56
Southwest	6.41	10.40
West	7.56	5.45
Northwest	9.65	1.19
North	12.26	5.84
Northeast	8.16	8.10
East	17.73	6.45

## References

[1] Hespenheide H.A. (1991). Bionomics of leaf-mining insects. Annu. Rev. Entomol..

[2] Maier C.T. (1983). Effect of the apple blotch leafminer (Lepidoptera: Gracillariidae) on apple leaf abscission. J. Econ. Entomol..

[3] Faeth S.H. (1991). Effect of oak leaf size on abundance, dispersion, and survival of the leafminer *Cameraria* sp. (Lepidoptera: Gracillariidae). Environ. Entomol..

[4] Kahn D.M., Cornell H.V. (1989). Leafminers, early leaf abscission, and parasitoids: A tritrophic interaction. Ecology.

[5] Stiling P., Simberloff D. (1989). Leaf abscission: Induced defense against pests or response to damage?. Oikos.

[6] Waddell K.J., Fox C.W., White K.D., Mousseau T.A. (2001). Leaf abscission phenology of a scrub oak: Consequences for growth and survivorship of a leaf mining beetle. Oecologia.

[7] Heads P.A., Lawton J.H. (1983). Studies on the natural enemy complex of the holly leaf-miner: The effects of scale on the detection of aggregative responses and the implications for biological control. Oikos.

[8] Fisher A.E.I., Hartley S.E., Young M. (2000). Direct and indirect competitive effects of foliage feeding guilds on the performance of the birch leaf-miner *Eriocrania*. J. Anim. Ecol..

[9] Brewer A.M., Gaston K.J. (2002). The geographical range structure of the holly leaf-miner. I. Population density. J. Anim. Ecol..

[10] Connor E.F. (1988). Cohort and death assemblage estimates of survival rates and causes of mortality in *Brachys ovatus* (Weber) (Coleoptera: Buprestidae). Am. Midl. Nat..

[11] Waddell K.J., Mousseau T.A. (1996). Oviposition preference hierarchy of *Brachys tessellatus* (Coleoptera: Buprestidae). Environ. Entomol..

[12] Fox C.W., Waddell K.J., Grokters F.R., Mousseau T. (1997). Variation in budbreak phenology affects the distribution of a leafmining beetle (*Brachys tessellatus*) on turkey oak (*Quercus laevis*). Ecoscience.

[13] Turnbow R.H., Franklin R.T. (1981). Bionomics of *Brachys tessellatus* in coastal plain scrub oak communities. Ann. Entomol. Soc. Am..

[14] Itaya Y., Sato K., Izumi Y., Hirayama H. (1981). Damage caused by *Trachys yanoi* in Arashiyama, Kyoto. For. Pests.

[15] Okuda M. (1983). Life cycle of *Trachys yanoi* Y. Kurosawa. Trans. Meet. Tohoku Br. Jpn. For. Soc..

[16] Hosoda R., Igarashi M., Ito K., Urano T. (1991). Capturing experiment of *Trachys yanoi*, a leaf miner of *Zelkova serrata*, by attractants. Annu. Rep. Kansai Res. Cen. FFPRI.

[17] Tsuchiya D. (1980). Ecology of *Trachys yanoi* and damage to *Zelkova serrata* caused by the insect in Tokyo. For. Pests.

[18] Ohmomo S., Fukutomi H. (2013). The Buprestid Beetles of Japan.

[19] Tsuchiya D., Kobayashi F., Taketani A. (1994). Trachys yanoi. Forest Insects.

[20] Kurata S. (1971). Illustrated Guide to the Important Forest Trees of Japan.

[21] Ohsawa M. (2012).

[22] Westcott R.L., Murray T.C. (2012). An exotic leafminer, *Trachys minutus* (L.) (Coleoptera: Buprestidae), found in Massachusetts, U.S.A.. Coleopts. Bull..

[23] Tomiczek C., Krehan H. (1998). The horsechestnut leafmining moth (*Cameraria ohridella*): A new pest in Central Europe. J. Arboric..

[24] Gilbert M., Guichard S., Freise J., Grégoire J.-C., Heitland W., Straw N., Tilbury C., Augustin S. (2005). Forecasting *Cameraria ohridella* invasion dynamics in recently invaded countries: From validation to prediction. J. Appl. Ecol..

[25] Straw N.A., Tilbury C. (2006). Host plants of the horse-chestnut leaf-miner (*Cameraria ohridella*), and the rapid spread of the moth in the UK 2002–2005. Arboric. J..

